# Engineered etoposide-prednisolone co-nanocrystals for synergistic antitumor efficacy enhancement

**DOI:** 10.1039/d5na00941c

**Published:** 2026-07-01

**Authors:** Panpan Ma, Abdelrahman Hassan, Sarah Diakhaby, Syed Nafis Shadman Ali, Luis Castillo-Henríquez, Marianne Bombled, Charlotte Izabelle, Libor Kostka, Tomáš Etrych, Rabah Gahoual, Brice Martin, Johanne Seguin, Nathalie Mignet, Yohann Corvis

**Affiliations:** a Université Paris Cité, CNRS, INSERM, UTCBS Lab 75006 Paris France yohann.corvis@u-pariscite.fr https://utcbs.u-paris.fr/en; b Department of Radiology, Lucas Center, 1201 Welch Road Stanford University Stanford CA USA; c Université Paris Cité, UAR3612 CNRS, US25 INSERM, Cellular and Molecular Imaging Facility 75006 Paris France; d Institute of Macromolecular Chemistry, Czech Academy of Sciences Heyrovského Náměstí 2 Prague CZ-162 06 Czech Republic; e Department of Neurological Surgery, Weill Medical College of Cornell University New York NY USA

## Abstract

The present work involves the development of new organic crystalline nanomedicines, namely, co-nanocrystals (co-NCs), that combine a chemotherapeutic agent with an anti-inflammatory active pharmaceutical ingredient (API), both in their nanocrystalline forms. These mixed systems were formulated to improve their anticancer efficiency. For this purpose, formulations composed of etoposide (ETO) and prednisolone (PRD) NCs were prepared. The co-formulations were optimized with the stabilizing agent poloxamer P407. The average size obtained was 191.9 ± 6.5 nm with a polydispersity index of 0.28 ± 0.09 and a zeta potential of −5.0 ± 0.7 mV for the fresh formulations. The drug loading was determined to be at 71.9% ± 0.6% for PRD and 84.1% ± 3.9% for ETO. The obtained co-NCs presented a long-term stability of at least 70 days after storage at 4 °C. Dialysis experiments showed that both APIs were released from the co-formulations following 1st-order kinetics. In addition, the NCs exhibited stronger antitumoral activities *in vitro* on Lewis lung carcinoma cells compared to the free ETO formulation or the ETO mono-NCs. Moreover, the anti-angiogenic properties tested on endothelial cells using capillary tube formation and the effect against cell migration analyzed by the scratch healing assay were enhanced by the ETO/PRD NCs co-formulation. The effect of the addition of PRD to the nanocrystalline formulation was shown by the greater reduction in inflammatory cytokine IL-6 levels *in vitro* when endothelial cells were incubated with the co-NCs as opposed to the mono-NCs. These results open the way for the preparation of stable co-formulations and better-tolerated combined therapies.

## Abstract 2

Original co-nanocrystal formulations for the colloidal stabilization of APIs and synergistic anticancer/anti-angiogenic efficiency on three different cell lines.

## Graphical abstract

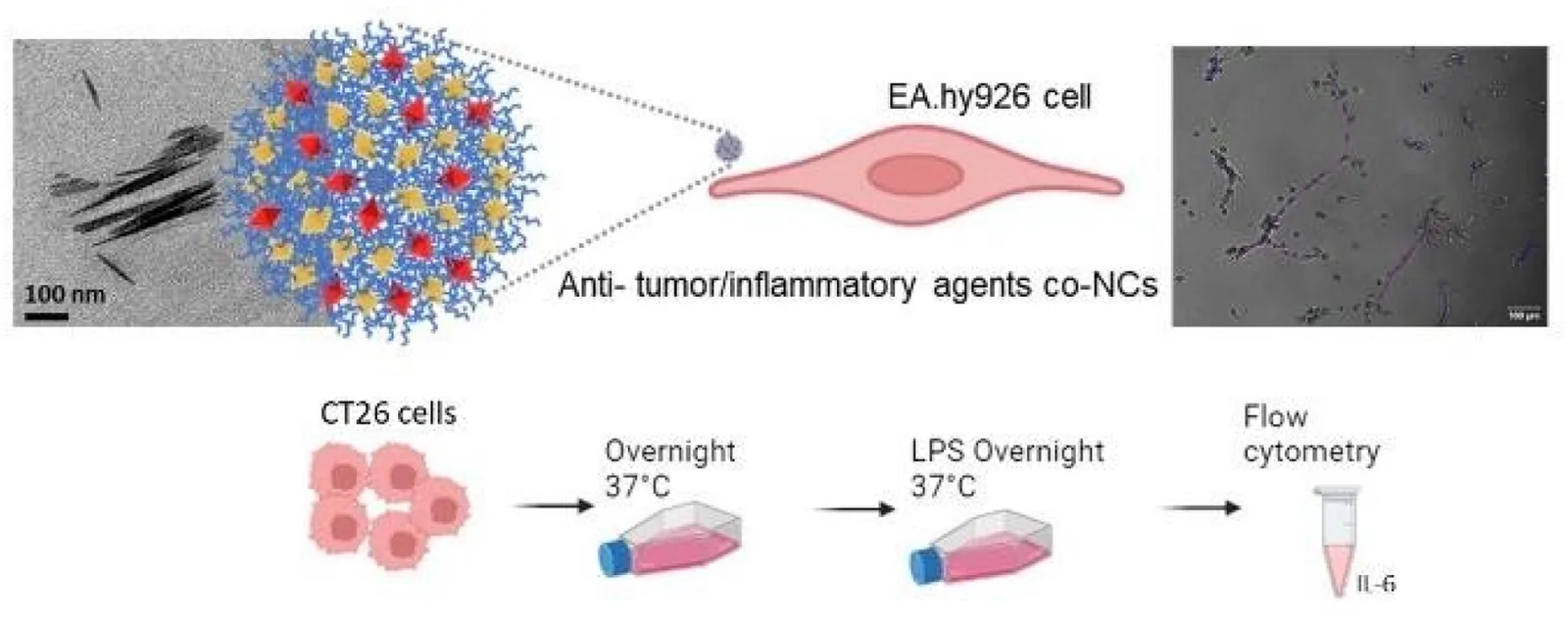

## Introduction

Cancer is a leading cause of death and a major obstacle to improving life expectancy all over the world, according to the Global Cancer Statistics 2020 report.^1^ Although modern oncology has developed different strategies and has made considerable advances in cancer treatment, such as stem cell transplantation,^2^ targeted therapy,^3^ and immunotherapy,^4,5^ chemotherapy remains one of the main tools in cancer treatment.^6^

However, 90% of the active pharmaceutical ingredients (APIs) currently being developed exhibit low aqueous solubility (classes II and IV of the biopharmaceutical classification system, BCS). Among the various solubilisation strategies, nanocrystallization is a promising technology, which has led to the formulation of 25 compounds on the market, *e.g.*, Invega Sustenna® (paliperidone palmitate), Ryanodex® (dantrolene sodium), and Cabenuva® (cabotegravir/rilpivirine).^7^ Due to their increased apparent solubility, nanocrystals exhibit enhanced pharmacokinetic^8–10^ and safety^11^ profiles, enabling their use for various routes of administration.^11–14^ Our group has worked on the nanocrystallization of anticancer drugs, such as etoposide (ETO),^8^ fisetin,^15^ and curcumin.^16^ Remarkably, ETO incorporation has been enhanced in terms of drug loading capacity, bioavailability, and anticancer efficiency due to its nanocrystallization.^8,15^ Nevertheless, although ETO has shown excellent preclinical anticancer activity, its subsequent clinical translation is constrained by its limited activity as a monotherapy agent.^17,18^

In this context, combination therapies, which combine an anti-inflammatory drug with a chemotherapeutic agent, represent an outstanding approach for enhancing therapeutic efficacy. Among the anti-inflammatory compounds, corticosteroids^19^ are commonly used to prevent or reduce the side effects of chemotherapy. Their abilities to modulate the tumor microenvironment,^20^ potentially reducing tumor cell migration and increasing their apoptosis,^21^ have been demonstrated to ultimately improve chemo-sensitivity.^22,23^ For instance, prednisolone (PRD, Fig. S1B), a synthetic cortisol compound, is frequently used to avoid immune reactions or to limit inflammation of certain tissues during organ transplantation.^24,25^ In 2023, a therapy based on PRD associated with olaparib and abiraterone for patients suffering from metastatic castration-resistant prostate cancer received FDA approval.^26^

Remarkably, PRD and ETO have already been combined with other drugs in several clinical studies, demonstrating the benefits of this combination in various applications, such as eyelid mass,^27^ high-risk acute lymphoblastic leukemia^28^ or acquired immunodeficiency syndrome-associated lymphomas.^29^ In 2019, Aoyagi Tetsuji's group showed that these combination therapy targeting inflammatory cells have the potential to attenuate lethal acute respiratory distress syndrome (ARDS).^30^ This was confirmed in 5 patients with COVID-19-associated ARDS whose treatment with ETO and low-dose PRD resulted in an overall favorable outcome.^31^

Based on these promising results, this work aims to evaluate the combination of the FDA-approved anticancer agent ETO^17,32^ with PRD as co-nanocrystals (co-NCs). This approach has not been proposed so far in the literature given that (i) solubility limitations are exacerbated in the formulation of combination therapies, as the two drugs to be delivered should be administered in the same medium, and (ii) the colloidal stability of PRD NCs is compromised by its low log *P* (1.62),^33^ as proven by only two top-down formulations reported in the literature.^34,35^ Indeed, the present work has proven that stable PRD NCs can only be obtained with good reproducibility if they are formulated with ETO using the solvent/antisolvent nanoprecipitation bottom-up approach optimized for the co-formulation engineering (Fig. 1).

**Fig. 1:**
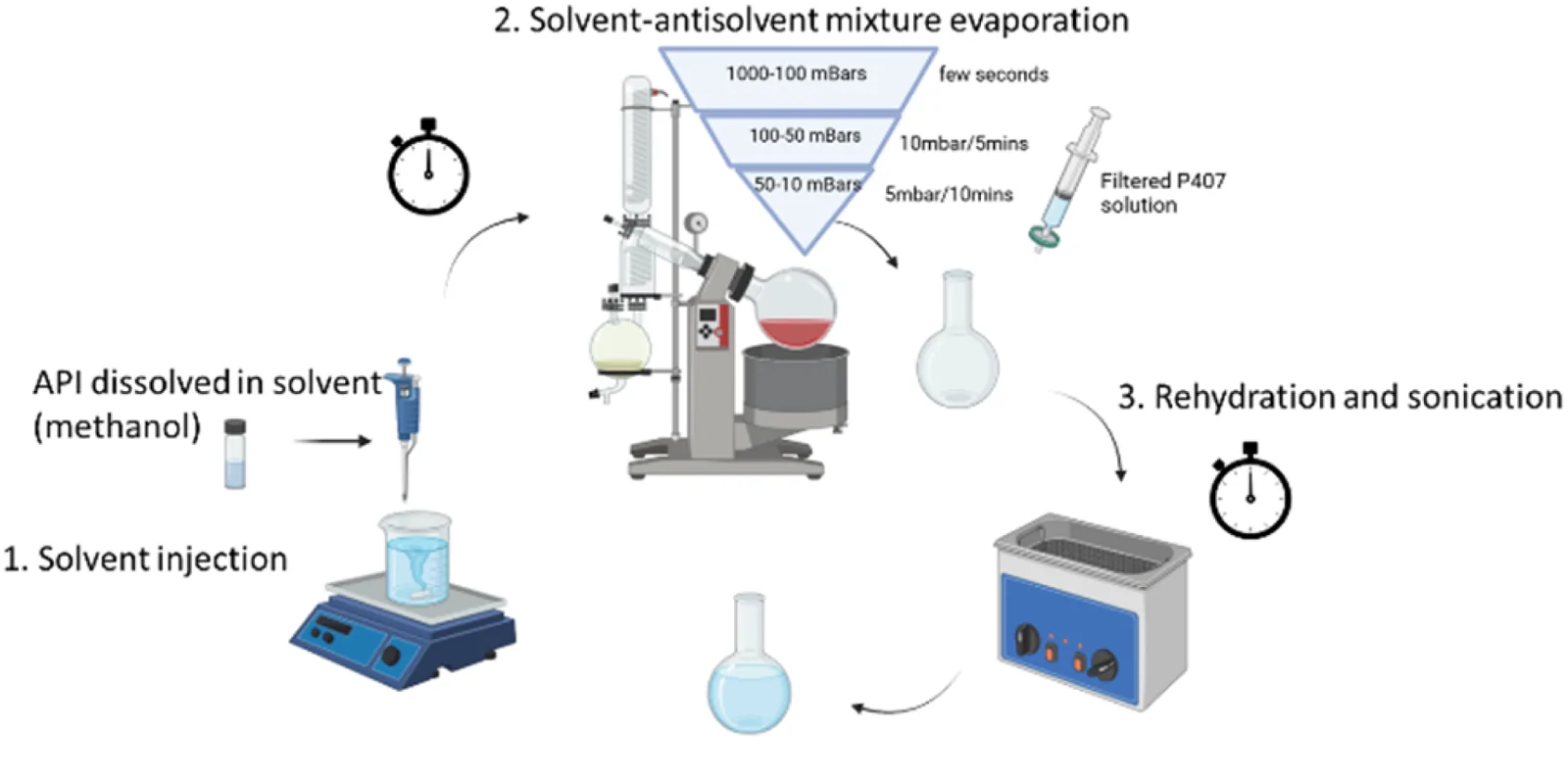
Nanocrystal preparation process adapted from Ma *et al.*^15^

Hence, a comprehensive physicochemical characterization of the different formulated NCs was performed using dynamic light scattering (DLS), scanning electron microscopy (SEM), transmission scanning electron microscopy (TEM), Fourier transform infrared (FT-IR) spectroscopy, and high-performance liquid chromatography (HPLC). The toxicity and the antimigratory, anti-angiogenic, and anti-inflammatory effects show the capacity of each drug to maintain its activity in the combined therapy.

## Material and methods

### Materials

PRD (Mw = 360.44 g mol^−1^, purity 99%, and CAS number: 50-24-8) and ETO (Mw = 588.56 g mol^−1^, purity 95–105%, and CAS number: 33419-42-0) were purchased from Sigma-Aldrich (MO, USA) and used without any further purification. ETO is a BCS class IV agent (*i.e.*, has low solubility and low permeability)^36^ that presents a log *P* of 0.73 (aqueous solubility at 25 °C: 80 mg L^−1^).^37,38^ Pluronic 407 (Poloxamer 407, 10000–14600 g mol^−1^/Ph. Eur.) was purchased from BASF (Ludwigshafen, Germany). Ultrapure methanol (MeOH) and dimethyl sulfoxide (DMSO) were purchased from Thermo Fisher Scientific Inc. (MA, USA) and Sigma-Aldrich (MO, USA), respectively. Deionized water (Milli-Q, filtered through a 0.2 µm membrane) was used for the present study. A 10 kDa regenerated cellulose membrane (Merck, Darmstadt, Germany) was used for filtrations prior to determining the drug loading of the NCs *via* HPLC experiments. ATCC-CRL-2638 CT26.WT colon carcinoma, mouse; ATCC-CRL-1642 LL/2 (LLC1), Lewis Lung Carcinoma, mouse (*Mus musculus*) (3LL), and ATCC-CRL-2922 EA.hy926, vascular endothelium, human (*Homo sapiens*) were purchased from LGC Standards SARL, Molsheim, France. Fetal bovine serum (S1400-100A) was provided by Dominique Dutscher SAS, Bernolsheim, France.

### Nanocrystals preparation

ETO and PRD NCs, as well as ETO/PRD co-NCs, were prepared by antisolvent precipitation. ETO and PRD (2.5 mg each) were co-dissolved in 3 mL of MeOH (*i.e.*, the solvent). The solution was sonicated for 10 min, and then slowly injected dropwise into 20 mL of Milli-Q water (*i.e.*, the antisolvent) under magnetic stirring for 30 min at 500 rpm. The solvent: antisolvent volume ratio was established to be 1.5 : 10 based on previous findings.^8,39,40^ The mixture was vacuum dried through different decompression stages, as reported.^15^ Then, the optimal proportion of the P407 aqueous solution (0.083% w/v) adapted from previous results^8,15^ was added into the dry powder for hydration and sonicated for 2 hours at a controlled temperature of ∼20 °C. The API:stabilizing agent mass ratios were set at 1 : 2 and 1 : 4 for the mono- and co-NCs, respectively.

### Storage of the NC samples

Excipient-free dry powders can be stored for several days at room temperature (RT) prior to the rehydration process.^39^ The NC suspensions were kept at 4 °C.

### Lyophilization of the NC suspensions

The NC suspensions were dried using a freeze-dryer (Alpha 2–4 LD plus, Christ®, Osterode am Harz, Germany). For this purpose, 0.5 mL samples of each mono- and co-NCs were transferred into Eppendorf® tubes and frozen at −80 °C overnight. Subsequently, the samples were placed in the freeze-dryer for 18 hours. The resulting powders were kept in a dry and lucifugal environment for further use. Physico-chemical characterization results from all extemporaneous formulations prepared up to one month after powder obtention were similar to those of the fresh NC formulations. The same trend was observed in the quantification of the active ingredients using HPLC.

### Dynamic light scattering and zeta potential evaluation

The hydrodynamic diameter of the NCs was determined using the translational diffusion coefficient obtained by dynamic light scattering (DLS) at 25 °C using the Stokes–Einstein equation.^41^ After 2 min sonication of the suspensions, 1 mL sample was transferred into a ZEN0040 cuvette and introduced into the Nano ZS (Nano ZS Malvern Panalytical, UK) device (*λ* = 633 nm, scattering angle: 173 deg., and fixed position of laser: 3 mm). The NC suspensions were also tested directly in the scintillation vial using a DLS-based VASCO KIN® (Nano-kin) instrument (Cordouan, France) (*λ* = 635 nm and scattering angle: 170 deg.). For the zeta potential analysis, 10 µL of a sodium chloride solution (NaCl, 1.01 M) was added to 1 mL of the NC suspension (final NaCl concentration: 10 mM) before sample introduction into a DTS1070 cuvette. Each measurement was carried out in triplicate at 25 °C.

### Transmission electron microscopy

The structure of the nanosuspensions was evaluated by TEM. For this purpose, a 200-mesh carbon formvar copper grid was covered with one drop (10 µL) of the NC suspension, blotted and dried at RT for two hours. The images were acquired using a Jeol 1400 transmission electron microscope (Jeol, Croissy-sur-Seine, France) operated at 120 keV and equipped with a RIO CMOS camera (AMETEK SAS, Elancourt, France).

### Nanoparticle tracking analysis

The NCs were diluted 4 times in ultrapure water and analyzed using a Malvern NanoSight NS 300 (software NTA version 3.4). The samples were injected into the inlet tubing using a syringe pump at a continuous rate (500 a.u. speed) for the initial setup to detect the NCs using a camera (sCMOS, Blue405 laser), and then, the speed was reduced to 50 a.u. for visualization. Samples were recorded in triplicate for 45 s by adjusting the camera level and screen gain to set the focus. LGC Standards SARL, MOLSHEIM, FRANCE.

### Stability study

The colloidal stability of the NCs after storage at 4 °C was assessed over time. For that purpose, the hydrodynamic diameter of each formulation was checked daily by DLS during the first week after production, and then every week for two months.

### Fourier transform infrared (FT-IR) spectroscopy

FT-IR experiments were performed to determine the structural changes in the samples due to chemical interactions by identifying the functional groups of the raw API and P407 raw powders as well as the NCs using a Shimadzu FT-IR spectrometer (Nakagyo-ku, Kyoto, Japan). The FT-IR spectra of the samples were obtained in the transmittance mode over a scan range of 4000 cm^−1^ to 400 cm^−1^. 45 spectral scans were conducted on each sample with a 4 cm^−1^ resolution. Note that the pure water or air FT-IR signal was subtracted from the signal measured for the NC suspensions or pharmaceutical powders, respectively.

### PRD and ETO quantification by HPLC

HPLC experiments were conducted using a Shimadzu DGU-20 A3R (Kyoto, Japan) system to quantify the PRD and ETO contents in the prepared co-NC suspensions, which allowed us to determine their drug loading and monitor the dialysis experiments (*cf.* dissolution study). An HPLC Nucleodur® column (endcapped 100–5 C_18_, 5 µm, 250 × 4.6 mm, Düren, Germany) was chosen as the stationary phase. Two different mobile phases were tested under isocratic conditions as eluents: (i) a mixture of acetonitrile (ACN) 20% v/v and tetrahydrofuran (THF) 10% *v/v* in Milli-Q water with a 0.5 mL min^−1^ total flow rate and (ii) a mixture of 1% *v/v* acetic acid and 41% *v/v* MeOH in Milli-Q water with a 0.75 mL min^−1^ total flow rate, adapted from already published works.^8,42^ Under the excitation wavelength of 242 nm, the calibration curve, representing the area under the curve (AUC) as a function of the drug concentration, was established from 2.9 to 911.3 µM and 0.1 to 288.8 µM for PRD and ETO, respectively. To determine the concentration of both drugs in the NC preparations, the nanosuspensions were completely dissolved in MeOH (1 : 9 v/v NC suspension:MeOH) and then filtered employing a 10 kDa filter for 10 min at 13 500 rpm in order to remove the P407 polymer. Finally, the filtered solution was 10-fold diluted, and 20 µL of this diluted sample was injected into the HPLC instrument under the above-mentioned conditions to determine the related concentration using the calibration curve.

The PRD and ETO drug loading were calculated using eqn (1):1
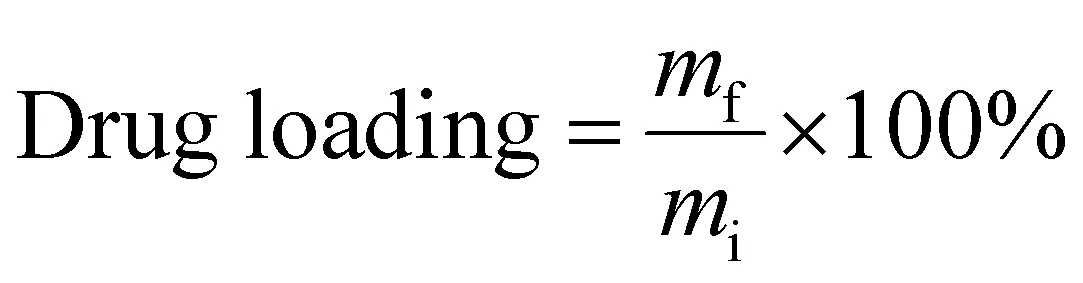
with *m*_f_ representing the mass of the drug determined from the NC formulations, and *m*_i_ the mass of the raw drug used to formulate the related preparation.

### Dissolution kinetics of the nanocrystals

*In vitro* release was assessed using the dialysis bag diffusion technique. 3 mL of the NC suspension was placed in a cellulose dialysis cassette (Slide-A-Lyzer™ G2, 10 kDa molecular weight cut off, Thermo Fisher Scientific Inc., Waltham, Massachusetts, United States). Then, the dialysis cassette was immersed, respecting sink conditions,^43,44^ in a compartment containing 300 mL of an HEPES-buffered saline (HBS) medium (20 and 150 mM HEPES and NaCl, respectively) in total at pH 7.4. The whole system was stirred at 50 rpm and maintained at 37 °C for one day. The receptor compartment was covered with an aluminum foil and parafilm to limit the evaporation of the continuous medium buffer. Aliquots (0.5 mL) were withdrawn from the receptor compartment at several time points from 15 min to 1 day, and the equivalent volume of fresh HBS was added to the continuous medium to maintain its overall volume after each sampling. Then, each sample was lyophilized as described above. The powder obtained afterward was dissolved in 0.5 mL of MeOH and then analyzed by HPLC as stated earlier. The cumulative release was calculated using the calibration curve determined for ETO and PRD, as described in the sub-section above.

### *In vitro* cytotoxicity and combined therapy assays on different cell lines

*In vitro* studies were conducted to evaluate the potential of the NCs on murine cancer and human endothelial cells. EA.hy926 and Lewis lung carcinoma 3LL cell lines were bought from the American Type Culture Collection (ATCC® CRL-2922 for the EA.hy926 cell line, and ATCC® CRL-1642 for the 3LL cell line; LGC Standards Ltd, Molsheim, France) and cultured at 37 °C in a 5% CO_2_-humidified atmosphere in the DMEM completed medium. The 3-(4,5-dimethylthiazol-2-yl)-2,5-diphenyltetrazolium bromide (MTT) test was performed as follows: Firstly, 100 µL of cell culture at a concentration of 100 000 cells per mL were seeded in some wells of a 96-well plate for 24 hours. Subsequently, 100 µL of the drug sample was added to each well of the plate at the concentrations ranging from 1.3 to 80.0 µM for PRD and 0.9 to 57.2 µM for ETO using serial two-fold dilutions. Finally, after 72 hours, the medium in each well was removed and replaced by the MTT solution in the culture medium (0.5 mg mL^−1^, 100 µL, M5655 Merck KGaA, Darmstadt, Germany) and incubated for 4 hours. To assess the living cells' activity, the culture medium was removed, and 100 µL of DMSO was added to each well plate (shaking at 150 rpm for 5 min). Then, the absorbance was measured at 560 nm using a microplate reader (Infinite F200 PRO, Tecan, Männedorf, Switzerland). The results were plotted as a percentage of viable cells as a function of the concentration of the incubated compound, calculated using the following equation:2
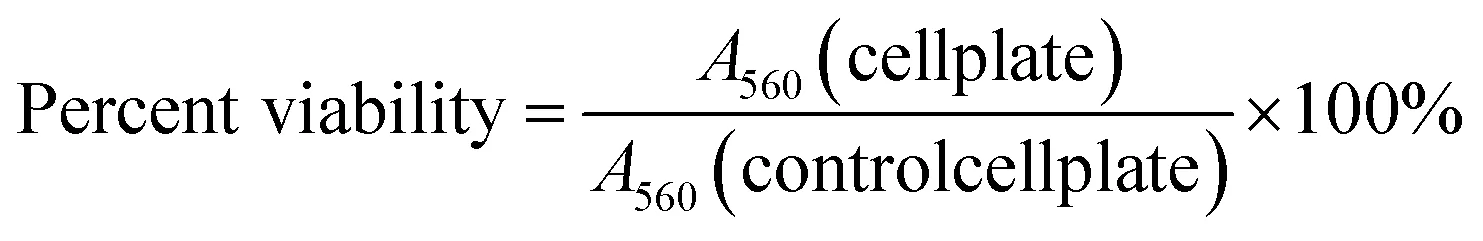
with *A*_560_ representing the absorbance measured at 560 nm. DMSO and poloxamer P407 were chosen as the control conditions for the free API and NCs, respectively.

Half maximal inhibitory concentrations (IC_50_) of each group were determined using GraphPad Prism Version 9.

### Angiogenesis assessment

Capillary tube formation was evaluated by an *in vitro* angiogenesis assay. As shown in our previous study,^15^ 50 µL of the growth factor-reduced Matrigel® solution (Catalog #354230, Corning®, Bedford, United States) was added to a 96-well plate and incubated for 30 min at 37 °C under 5% CO_2_. Then, the EA.hy926 endothelial cells (100 000 cells per mL) were incubated in a 100 µL of serum-free medium complemented with the basic fibroblast growth factor (10 ng mL^−1^, Catalog #610072, BD Biosciences, San Jose, USA) in the presence of 10 µM of the API for the NCs and free API systems and the equivalent P407 or DMSO content. After 24 hours exposure, the tube number and their length were observed at a magnification of ×100 with a Zeiss Axiovert 135 microscope (Carl Zeiss France, Le Pecq, France) and analyzed using the angiogenesis analyzer program from the Fiji software. The data were processed with GraphPad Prism version 9 from the micrograph analysis of three independent experiments.

### Evaluation of the angiogenesis effect of the vascular endothelial growth factor (VEGF) measurement

EA.hy926 endothelial cells (15 000 cells per mL) were plated into 6-well plates and incubated for 24 hours at 37 °C under 5% CO_2_. After that, the cells were exposed to either NCs, free API, P407, or DMSO dispersed/diluted in DMEM to reach an optimized concentration of 10 µM of the API for the NCs and free API systems and the equivalent P407 or DMSO content for 72 hours. Subsequently, the medium was collected, and VEGF was measured using a Human VEGF ELISA Kit (KHG0112, Thermo Fisher Scientific, Illkirch, France) based on the manufacturer's instructions. The data were treated with GraphPad Prism version 9, and the results were represented as %VEGF decrease compared with the control groups (NCs/P407 and free API/DMSO).

### Cell migration assay

EA.hy926 endothelial cells (200 000 cells per mL) were plated into a 96-well plate for 24 hours for confluence, and then a 100 µL pipette tip was used to introduce a wound area. The images were taken immediately from each well at a magnification of 100× (time 0 h) using a light microscope (Phase Contrast ELWD 0.3, Nikon, Tokyo, Japan). After 24 and 48 hours of incubation, digital pictures of the wound areas were taken. Cell migration was determined by the wound area at 0 h, 24 h and 48 h. All experiments were conducted in duplicate for each product and repeated 3 times. At least 3 views of the photo were taken across the field of view for each well. Area values were measured using the wound healing size tool program from the Fiji software, and the results were obtained as a percentage of the control group using the following equations:3
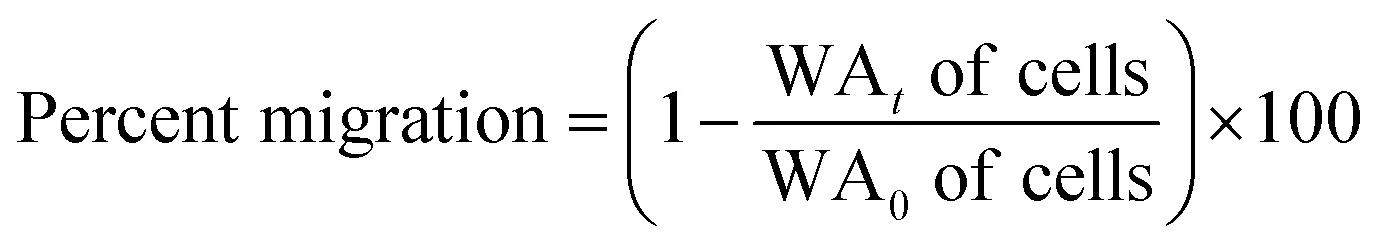
with WA_0_ representing the wound area at 0 h (pixels^2^), and WA_*t*_ is the wound area at *t* h (pixels^2^).

Detection of *in vitro* IL-6 inflammatory cytokine by flow cytometry and ELISA in response to the ETO/PRD co-nanocrystals 1 × 10^6^ CT26 cells per well were plated in a 48-well plate in duplicate and treated with the following formulations for 24 hours at 37 °C, 5% CO_2_: poloxamer 407 at 0.83 mg mL^−1^, DMSO at 0.833% (v/v), free etoposide at 35.4 µM, free PRED at 57.8 µM, free etoposide and prednisolone at 35.4 µM and 57 µM, respectively, etoposide nanocrystals at 35.4 µM, etoposide and prednisolone nanocrystals at 35.4 µM and 57.8 µM respectively. Cells were stimulated with a 1 µg mL^−1^ final concentration of LPS (Merck 437650) for another 24 hours at 37 °C, 5% CO_2_. Brefeldin A (BioLegend 420601) was added overnight within LPS incubation for flow cytometry analysis. Similar experiments were carried out at the same time without the brefeldin A treatment to measure IL-6 in the supernatant, which was kept at −20 °C prior to analysis.

Treated CT26 cells were incubated with 7-AAD (BioLegend 640934) for 15 min at room temperature, then fixed and permeabilized (BD Cytofix/Cytoperm 554714) for 20 min at 4 °C. Cells were stained with APC anti-IL-6 (BioLegend 504507) for 30 min at 4 °C and washed with FACS buffer (5% FBS and 0.067 mM EDTA in PBS). Flow cytometry analysis was performed using a Cytek Aurora Spectral Analyzer (RRID: SCR_019826), and the data were analyzed using the FlowJo software (TreeStar, version 10.10).

The treated CT26 cell supernatant was collected, and IL-6 levels were evaluated using a cytokine-specific enzyme-linked immunosorbent assay (ELISA) kit. The mouse IL-6 production was measured using ELISA (Mouse DuoSet ELISA kits DY008B and DY406-05, R&D Systems) according to the manufacturer's instructions.

### Statistical and fitting analysis

Statistical analysis was done using GraphPad Prism version 9 with a two-way analysis of variance (ANOVA) with a Bonferroni or a Kruskal–Wallis with Dunn's multiple comparison analysis. For the *in vitro* cytotoxicity studies, the percentage of viability as a function of log (concentration) and the non-linear transformation with a sigmoidal dose-response (variable slope) were applied. The release kinetics profile was fitted with the nonlinear regression fitting from GraphPad Prism version 9. Statistical significance was represented by *P < 0.05, **P < 0.01, ***P < 0.001 and ****P < 0.0001.

## Results and discussion

### Formulation and physicochemical characterization

The preparation method developed in our laboratory^15^ was transposed to the production of the PRD NCs and ETO/PRD co-NCs.

As for the ETO NCs, various P407 concentrations ranging from 5.1 × 10^−3^% to 83.3 × 10^−3^% (w/v) were screened to ensure adequate colloidal stability. The concentration of 83.3 × 10^−3^% (w/v) was selected considering the lowest hydrodynamic diameter and polydispersity index obtained upon long-term storage (Fig. S2A).

Assuming that the optimal P407 concentration corresponds to minimal free polymer or even micelles in the solution, the drug-to-polymer molar ratio was estimated to be 30 : 1. Lower and higher polymer concentrations have proven to exhibit either insufficient surface coverage or significant micellization, respectively. In both cases, ineffective steric stabilization results in the aggregation of the nanosized drug. It should be noted that the volume of the antisolvent and the mass of the stabilizer used for the co-NC preparations are twice as large as those used for the mono-NCs, in order to maintain the same solvent-to-antisolvent volume ratio of 1.5 : 10 and the API to stabilizer mass ratio of 1 : 2.

Furthermore, particle size plays an important role in the cellular uptake, biodistribution, drug release kinetics, and toxicity of nanomaterials.^45^Fig. 2 shows the hydrodynamic diameter determined using classical DLS and VASCO KIN® (Fig. 2A) and the polydispersity index (Fig. 2B) of the co-NCs, which were measured over time from d0 to d70. The co-NCs showed physical stability for at least 70 days after storage at 4 °C. Noteworthily, the hydrodynamic diameter of the ETO NCs was not significantly different from that of the co-NCs (Fig. 2C and Table S1).

**Fig. 2:**
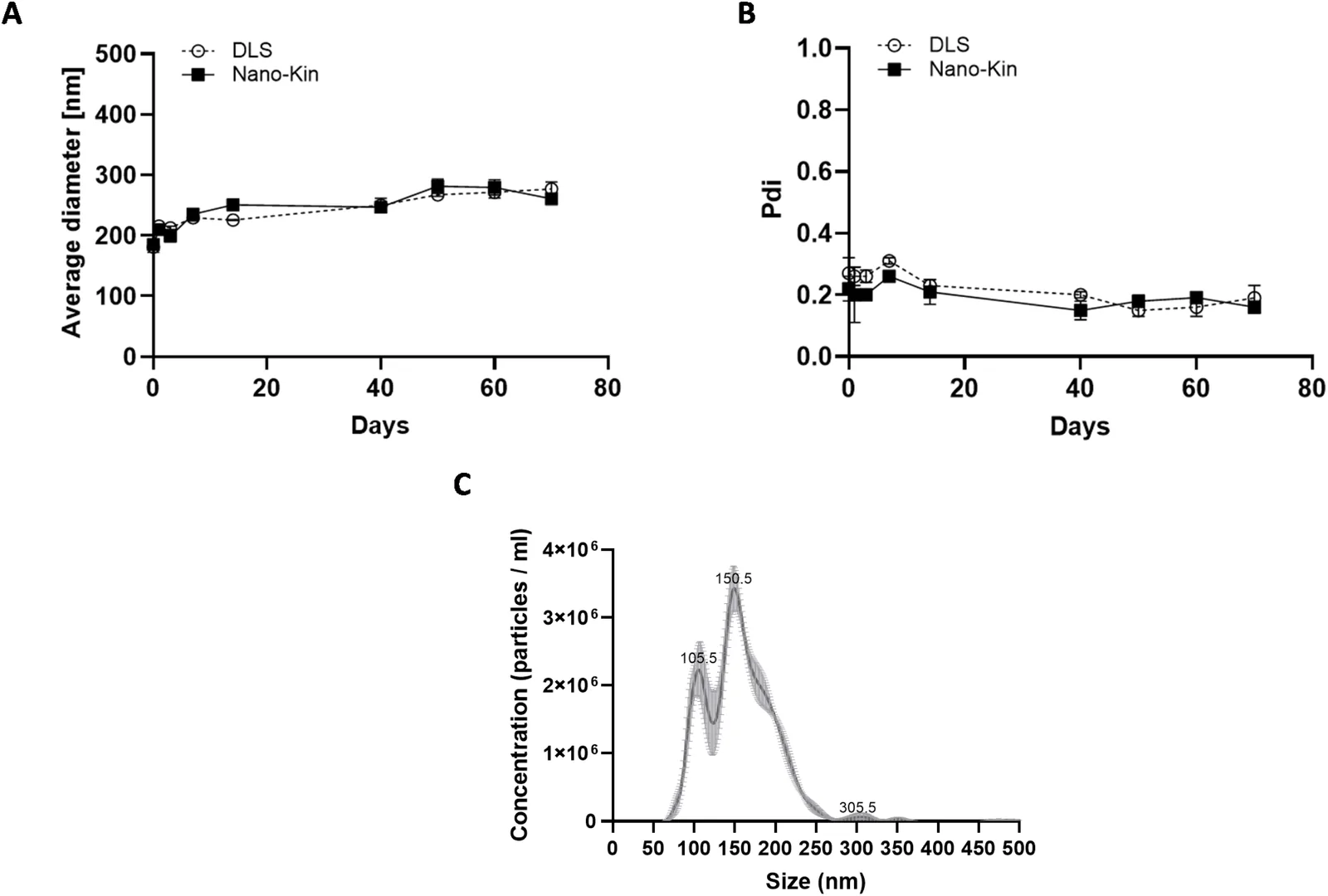
Long-term stability evaluation in terms of the hydrodynamic diameter (A) and polydispersity (B) for the ETO/PRD co-NCs stabilized with P407 obtained using classical dynamic light scattering (DLS) and VASCO KIN® (Nano-kin). (C) Nanoparticle tracking analysis of the size distribution of 4-fold diluted ETO NCs.

As for the PRD NCs, although this work demonstrates better stability compared to the preparations obtained by the H69 process, which became micron crystals after being stored at RT for about 4 hours,^35^ the present PRD NCs also show aggregation over time. However, the successful formulation of the combination therapy suggests that the presence of ETO aids in the stabilization of the PRD NCs due to specific interactions.

Moreover, as previously documented, DLS measures fluctuations in scattering intensity to determine the average hydrodynamic diameter, but its accuracy is notably influenced by the presence of larger particles due to their significant contribution to the overall scattering.^46^ Consequently, nanoparticle tracking analysis (NTA) was used for orthogonal characterization^47^ as it is performed on a particle-by-particle basis, enabling the resolution of larger particles or aggregates that are not resolved by DLS (Fig. 2C).

For a deeper understanding of the NCs organization in the formulated suspensions, the surface morphology and size of the particles were observed using various electron microscopy techniques: SEM and TEM. Raw PRD particles appear as rod-shaped crystals on a micron scale before and after the solvent/antisolvent precipitation process, which is confirmed by SEM (Fig. 3A and B). After evaporating the water from the PRD NCs or co-NCs suspensions, SEM visualization of the resulting powders confirmed a reduction to the nanometer scale with aggregated NCs (Fig. 3C and D). The TEM image of the co-NCs reveals that the addition of the stabilizing agent P407 results in monodispersed nanoparticles in water (Fig. 3E). The ETO/PRD co-NCs appear as a rod-shaped crystal, similar to our observation with the ETO mono-NCs,^15^ demonstrating that the shape of the NCs is not affected by the multidrug formulation. The average size of the ETO/PRD co-NCs obtained from the TEM images, *i.e.* 171 ± 10 nm, has slightly smaller size than the mean hydrodynamic diameter measured with DLS (Fig. S2B) and in accordance with the NTA results, which can be explained by the fact that the DLS cumulant analysis should be applied to spherical nanoparticles to obtain an accurate size. However, one can see from the TEM images that the NCs are not spherical, which might explain this slight discrepancy.^15^ In addition, the shaded parts in Fig. 3E may be the stabilizer P407 coating on the crystal surface.

**Fig. 3:**
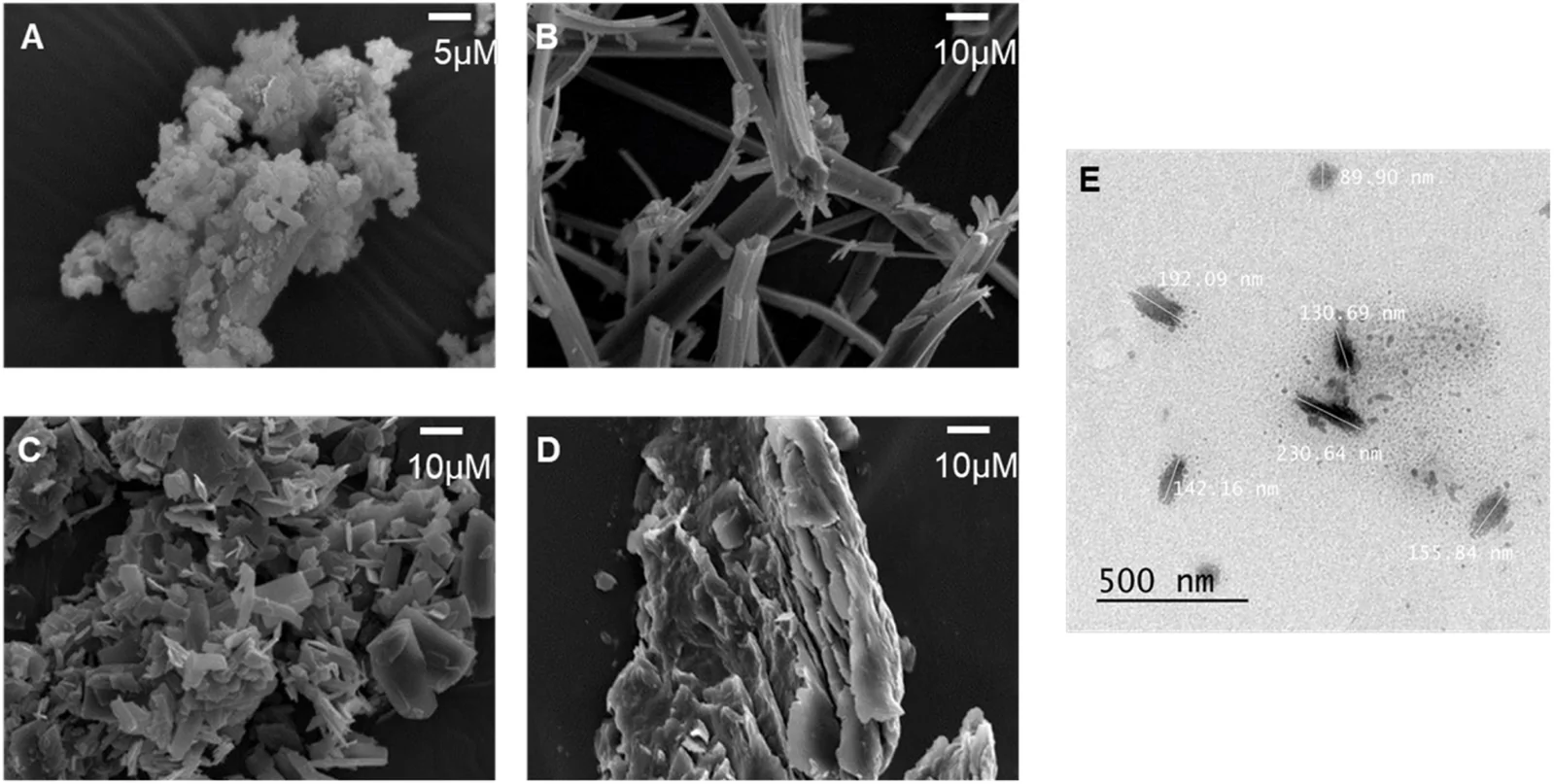
SEM images of aggregated raw PRD (A), PRD after organic solvent/water evaporation (B), PRD NCs after water evaporation (C), and the ETO/PRD co-NCs after water evaporation (D). TEM image (E) of the ETO/PRD co-NCs.

To gain a better understanding of the specific polymer-NC interactions, the PRD raw powder, poloxamer P407 raw powder, and the co-NCs were tested by Fourier transform infrared (FT-IR) spectroscopy (Fig. 4). The characteristic absorption bands of the PRD raw powder showed the OH signal involved in intermolecular bonding at 3200–3500 cm^−1^. C–H stretching vibration (alkene) is present at 2890 cm^−1^. Two carbonyl stretching peaks appeared as a very strong band at 1708 cm^−1^ and 1654 cm^−1^. Notably, the characteristic absorption peaks of PRD after organic solvent/water evaporation remained unchanged, indicating that the evaporation process did not affect the structure and properties of the substance. In addition, P407 showed principal absorption peaks at 2879 cm^−1^ (aliphatic C–H stretch), 1342 cm^−1^ (in-plane O–H bend), and 1099 cm^−1^ (C–O stretch). Interestingly, the co-NCs exhibited a reduction in the 3200–3500 cm^−1^ (OH group) and 1099 cm^−1^ (C–O stretch) regions, indicating a covalent bond interaction between PRD and P407. Additionally, an OH band shift was noted, which may suggest the existence of hydrogen bonding.

**Fig. 4:**
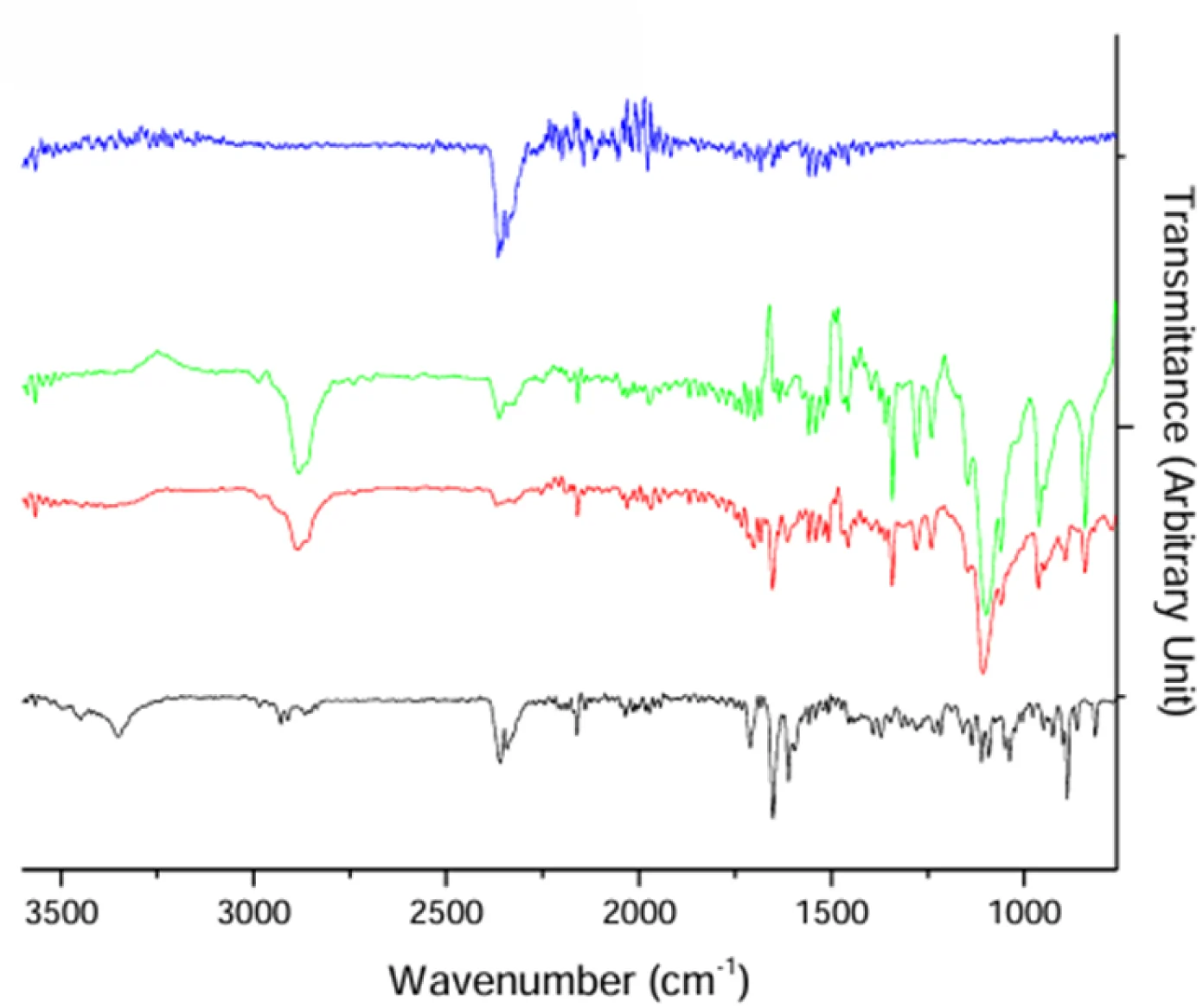
FT-IR spectra obtained for the PRD raw powder (black), poloxamer P407 raw powder (green), PRD after organic solvent/water evaporation (red), and co-NC suspension (blue). The intensity of the co-NC suspension spectrum showed a 3.15-fold increase, and the curves were shifted for clarity.

Overall, it can be assumed that a possible interaction could occur between the hydroxyl or carbonyl group of the nanocrystallized PRD and the hydroxyl group of P407. The effectiveness of physically coated nanoparticles with the copolymer has been demonstrated in fisetin/polymer nanocrystals^15^ and oxazolidine/polymer nanoparticles^48^ as well.

### HPLC prednisolone and etoposide quantification

The aim of this section was to develop and optimize a simple and cost-effective HPLC method for the detection of both drugs. Hence, we compared different mobile phases. As shown in Fig. S3 and Table S2, the retention time and chromatographic intensity of free PRD and free ETO are particularly close and difficult to distinguish when the mobile phase is 70% water/20% ACN/10% THF. On the contrary, when we used the mobile phase containing 1% acetic acid and 41% MeOH in Milli-Q water with a 0.75 mL min^−1^ total flow rate, the retention time and chromatographic intensity of PRD and ETO were clearly separated (Fig. S3C). The latter mobile phase represents a good compromise in terms of sensitivity and peak separation for the 242 nm wavelength of absorption. PRD and ETO were found to have retention times of 50.3 ± 0.8 min (*n* = 14) and 30.4 ± 0.7 min (*n* = 16), respectively, with the optimized mobile phase. The calibration curves for the AUC as a function of ETO and PRD concentrations were *y* = 11 143 × [ETO]_µM_ (*r*^2^ = 0.9995) and *y* = 16 310 × [PRD]_µM_, *r*^2^ = 0.9992, respectively.

### Dissolution study of the nanocrystals and the evaluation of the release model

Sustained drug release from NCs is essential for chemotherapy therapeutic efficiency. The *in vitro* release of PRD and/or ETO from the mixed or individual NCs was monitored using dialysis in HBS buffer solutions (pH 7.4), simulating physiological conditions. It was found that the ETO/PRD co-NCs or mono-NCs achieved a prolonged drug release within at least 3 h, as presented in Fig. 5. We further found that PRD and ETO were released simultaneously from the co-NCs, following a 1st-order kinetics (Fig. S4). On the one hand, the cumulative release of ETO and PRD from the co-NCs followed approximately the same profile, reaching 76% and 82% at 6 hours and 84% and 88% at 24 hours, respectively. On the other hand, the release was higher in both cases in the mono-NC preparations (Table 1). As presented in Table 1 and Fig. S4, the NC formulations exhibited drug loading ranging from 72% to 97%, and the ETO NCs had a much higher drug loading than the PRD NCs. In addition, these results indicated the influence of the nature and number of APIs on the drug loading. The single drug-containing NCs showed a higher drug loading than the dual drug-containing NCs. Besides, the mixed NCs strategy demonstrates its feasibility as an effective approach for the co-delivery of combined therapeutic agents.

**Fig. 5:**
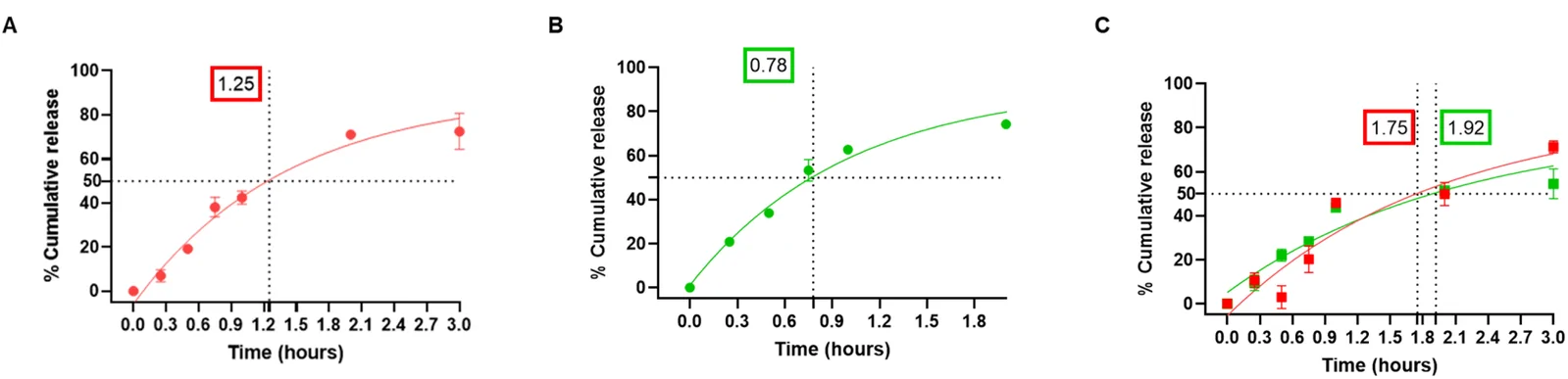
Release kinetics profiles of ETO (A) and PRD (B) from the mono-NCs as well as ETO (red) and PRD (green) released from the co-NCs (C) in HBS incubated at 37 °C under shaking (*n* = 3). The time required for drug accumulation to reach 50% (EC_50_) for each API from the corresponding formulation is indicated in the square inset.

**Table 1:** Cumulative release and yield values for the different NC formulations, value ± SD, *n* = 3

Formulation	Cumulative release (%)		Yield (%)
	6 h	24 h	
ETO NCs	88.9 ± 1.1	91.2 ± 1.3	82.6 ± 2.1
PRD NCs	90.0 ± 1.1	97.7 ± 4.5	97.1 ± 3.9
PRD in ETO/PRD co-NCs	76.2 ± 3.2	83.6 ± 6.6	71.9 ± 0.6
ETO in ETO/PRD co-NCs	82.3 ± 6.7	88.3 ± 1.9	84.1 ± 3.9

### MTT evaluation performed on endothelial and 3LL tumor cells

To investigate whether the ETO/PRD co-NCs enhanced anticancer efficiency, we used the MTT assay to measure cell viability with various concentrations of formulations on 3 different cell lines, namely, 3LL and CT26 cancer cells as well as EA.hy926 endothelial cells. In clinical studies, etoposide is used for the treatment of non-small-cell lung cancer. Although 3LL is not the most appropriate cell line, tests carried out with the latter provide more insights into the treatment efficiency of the nanoparticles formulated. As shown in Fig. 6, all groups showed dosage-dependent inhibitory effects on cells, while the NC groups showed a slightly superior inhibition on the cell proliferation than the free ones after 72 hours of incubation. The 3LL cell line was more sensitive to ETO NCs than the CT26 and EA.hy926 cell lines, with an IC_50_ value of 0.7 ± 0.1 compared to 3.7 ± 0.3 and 31.6 ± 1.0 µM, respectively (Table 2). Moreover, 1.0 ± 0.1 µM ETO in the free ETO system was required to kill 50% of the 3LL cells, which was 2 times more than that required in the ETO/PRD co-NCs (0.5 ± 0.1 µM). These results indicate the efficiency of the ETO/PRD nanocrystalline co-formulation compared to the free drug-related mixture for all the cell lines that have been evaluated. These findings confirm better internalization of the drugs when they are formulated as NCs, which is consistent with the trend of previous studies, such as celastrol/doxorubicin nanocrystals^49^ and co-delivery of etoposide/curcumin by a lipid nanoparticulate drug delivery system.^50^ No significant difference in the IC_50_ data was observed between the free ETO, the ETO NCs and the ETO/PRD co-NCs systems regarding the CT26 cell line. Noteworthily, ETO is less toxic to EA.hy926 endothelial cells than to cancer cells, which allows for better monitoring of the PRD impact on cell tube formation, vascular endothelial growth factor (VEGF) expression, and the antimigratory effect.

**Fig. 6:**
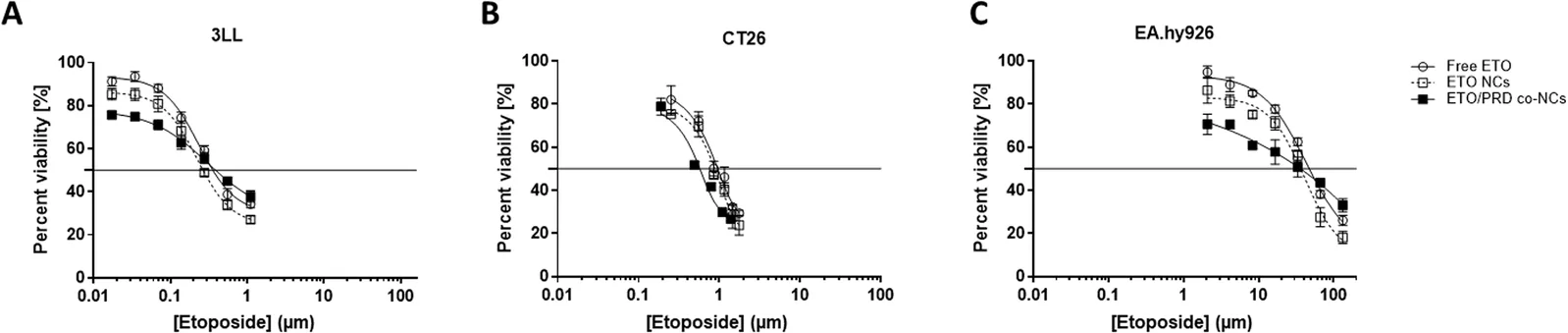
Effect of the ETO NCs and ETO/PRD co-NCs on the viability of the tumor 3LL (A), CT26 (B), and EA.hy926 endothelial (C) cells after 72 hours of incubation. Mean ± SEM with *n* = 3 independent experiments.

**Table 2:** Results obtained with 3LL and EA.hy926 cells after 72 hours of incubation with different NCs. IC_50_ values, given as ETO concentration, were calculated using the Sigmoidal dose-response (variable slope) equation with the GraphPad software. Mean ± SEM with *n* = 3 independent experiments, Kruskal–Wallis with Dunn's multiple comparisons test

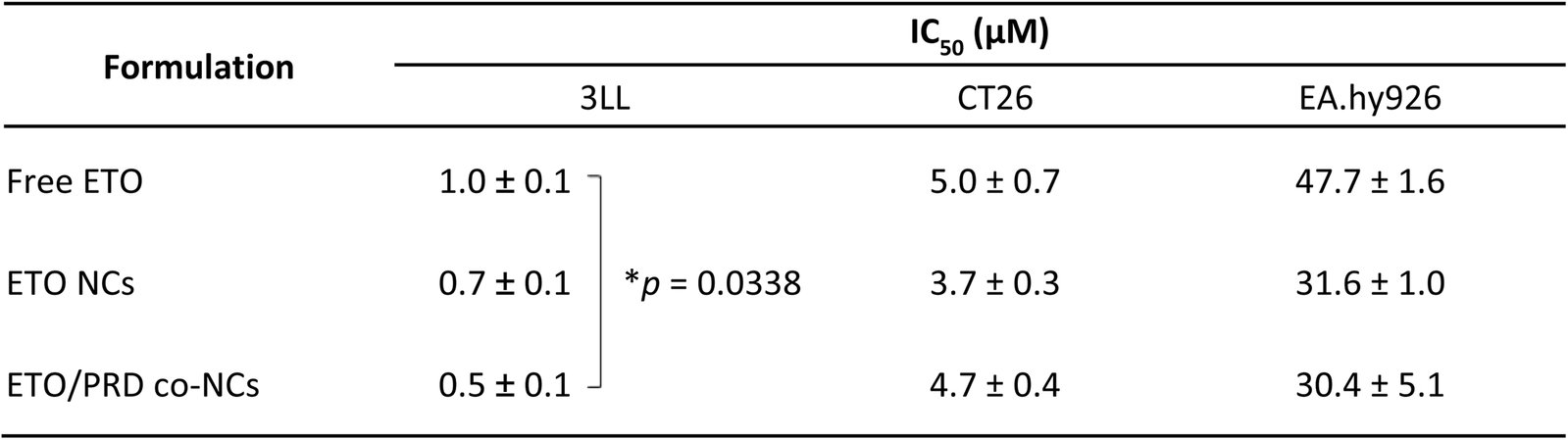

### Endothelial cell tube formation (EA.hy926 morphogenesis on matrigel)

To investigate whether the ETO/PRD co-NCs have an effect on angiogenesis, EA.hy926 cells were cultured in a Matrigel® environment, allowing the analysis of the segments and branches of the pseudo-vessels formed (Fig. 7A–C). The images show a clear effect on endothelial cells incubated with the ETO/PRD co-NCs formulation. The white double-headed arrow in the Fig. 7B photograph is an example of segment disruption, which leads to the regression of a capillary-like motif. This, in turn, results in mesh extension characterized by a decrease in the mesh area and total length values. After analysis of the total mesh area, total length and total segment length using the angiogenesis analysis program of the Fiji software, only the ETO/PRD co-NC formulation is statistically different for the total length compared to the PRD mono-NCs (Fig. 7D), while the total mesh areas are not impacted (Fig. 7E). These results confirm the synergistic effect of ETO and PRD when they are co-formulated, which is consistent with previous studies showing that nanotechnologies combined with anti-angiogenic therapies have a significant effect.^51^

**Fig. 7:**
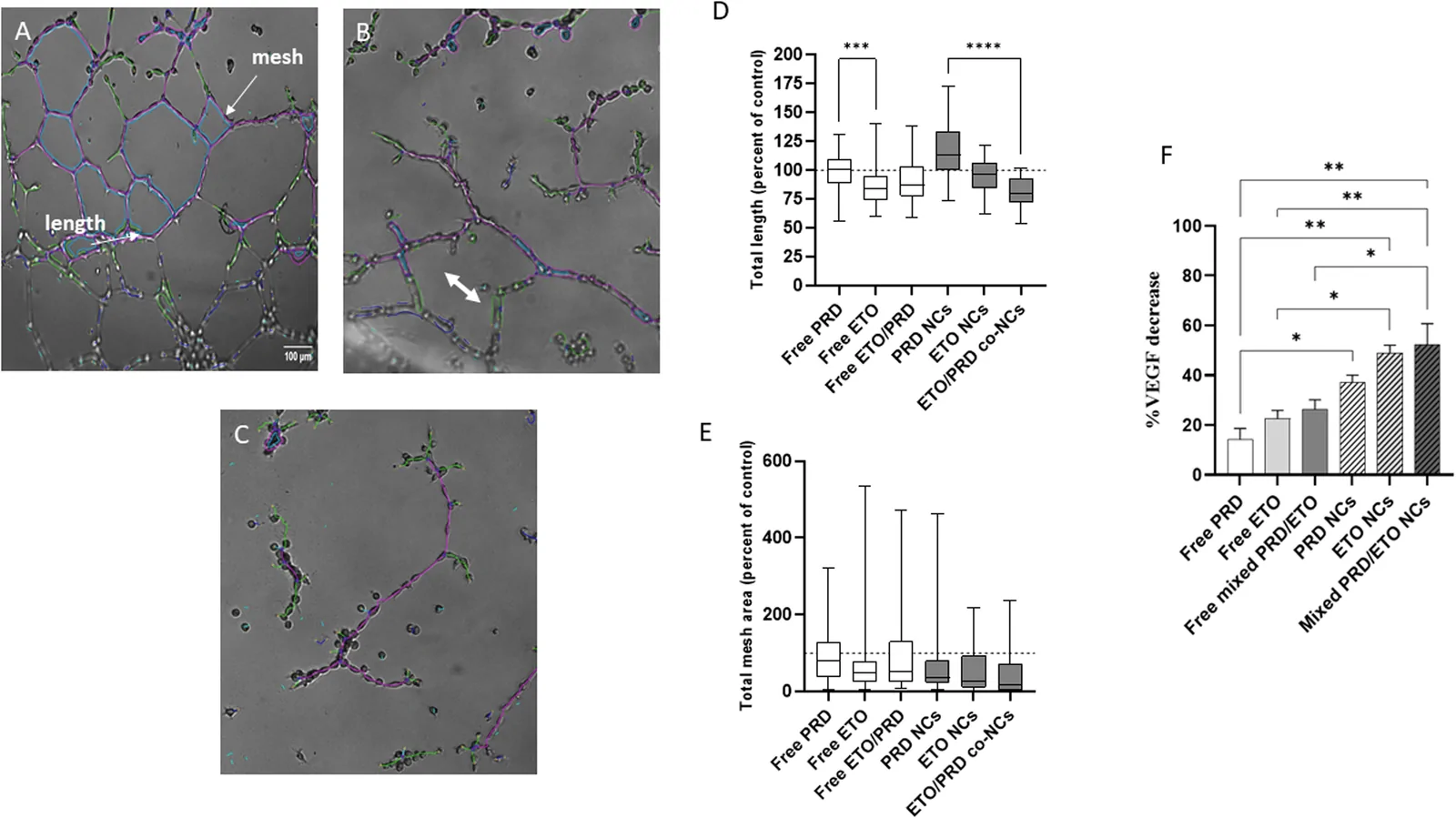
Images of EA.hy926 endothelial cells (×100) cultured in a Matrigel® environment after 24 h incubation with control cells only (A), 10 µM free mixed PRD/ETO (B) and 10 µM ETO/PRD co-NCs (C). Scale bar, 100 µm. Organization of pseudovessels: branches, segments and meshes are indicated by white arrows in Panel A. (D and E) Corresponding total length and mesh area obtained after image analysis. (F) VEGF levels expressed as a decreased percentage in EA.hy926 cells, 3 days after treatment with NCs and free drug at 10 µM. Data were processed from the micrographs using GraphPad Prism version 9 and represented as the mean ± S.E.M. of three independent experiments, Kruskal–Wallis test: *P < 0.05, **P < 0.01, ***P < 0.001, and ****P < 0.0001.

### Evaluation of the angiogenesis effect: VEGF measurement in the supernatant

Vascular endothelial growth factor is a crucial growth and survival factor for endothelium.^52^ VEGF is a signaling protein produced by many cell types that stimulates angiogenesis. To investigate whether the ETO/PRD co-NCs reduce angiogenesis by downregulating endothelial-secreted growth factors, VEGF levels were assessed in the supernatant of EA.hy926 cells incubated with 10 µM of the different formulations using an ELISA kit. As shown in Fig. 7F, the nanocrystal form significantly reduces the VEGF levels compared to the free drugs, *e.g.*, mixed ETO/PRD reduced VEGF by around 20%, while ETO/PRD co-NCs by around 50%. It is well known that corticosteroids such as PRD inhibit the platelet-derived growth factor-induced transcription of VEGF mRNA and secretion of VEGF protein.^53^ Moreover, it has been shown that ETO can inhibit VEGF secretion in U87 human glioblastoma and Lewis lung carcinoma cell lines by 51% and 36%, respectively.^17^ Consequently, we can deduce that the ETO/PRD co-NC formulation induced better internalization of the APIs compared to the free drug mixtures on the one hand and the mono-NCs on the other hand owing to its co-nanocrystalline form.

### Antimigratory effect of the ETO/PRD combination on EA.hy926 cells

A wound-healing assay is a way to replicate the process of cell migration that occurs in a living organism during the development of cancer, especially in the context of metastasis (Fig. 8). As shown in Fig. 8A, the control cells migrated and completely filled the wound area after 48 hours, whereas the ETO/PRD co-NC treatments clearly inhibited cell migration, which is consistent with the expected role of corticosteroids in inhibiting endothelial cell migration.^54,55^ In Fig. 8B, a quantitative analysis of the percentage of cell migration was performed. At 48 hours, both the control and ETO NCs had completely covered the wound area, with 100% migration for both groups. At this time point, the PRD NCs and ETO/PRD co-NCs exhibited 25.3% and 13.1% cell migration, respectively. However, only the combination of the two drugs significantly reduced migration compared to the control.

**Fig. 8:**
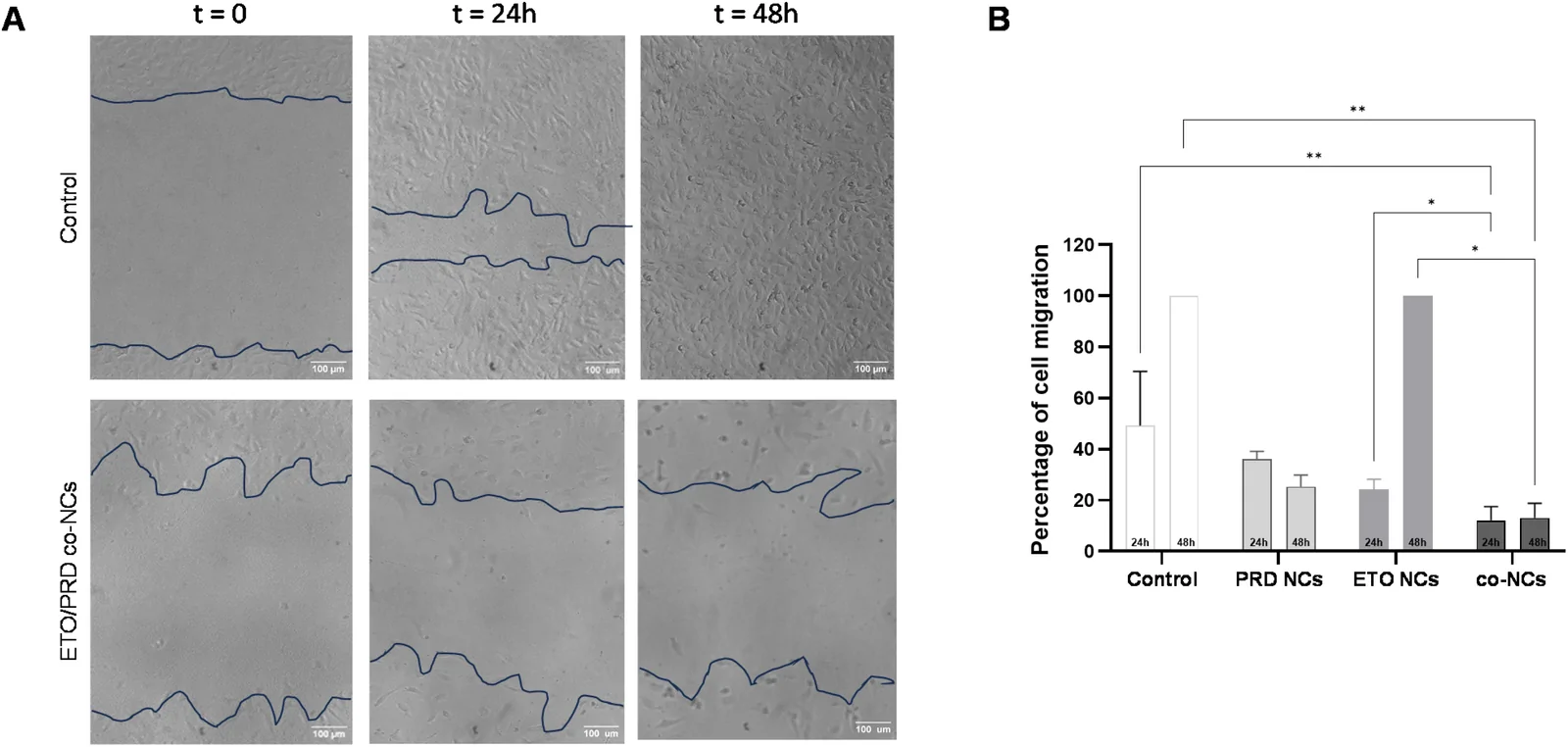
(A) Representative phase-contrast microscope images of the scratch wound-healing assay performed with EA.hy926 endothelial cells at 0, 24 and 48 hours, scale bar, 100 µm. (B) Corresponding quantitative cell migration monitored by the rate of cells moving towards the scratched area over time (at 24 and 48 hours) as a percentage of the area at *t* = 0. Data were processed using GraphPad Prism version 9 and represented as the mean ± S.E.M. of three independent experiments, 2-way ANOVA with Tukey's multiple comparisons test *P < 0.05 and **P < 0.01.

### Effect of ETO/PRD co-NCs on IL-6 production in CT26 cells

In our study, we were interested in checking the effect of PRD on the inflammatory IL-6 cytokine levels in CT26 cells (Fig. 9). The addition of PRD in the free or the co-NC form did not increase the percentage of tumor cell death, as previously evaluated (Fig. 9A). However, looking at IL-6 levels in the same cell line, the addition of PRD significantly decreased IL-6 cytokine expression, especially for ETO/PRD co-NCs (Fig. 9B and C), indicating that the synergistic effect of the co-formulation antitumoral property emphasized in the frame of the present study may be due to the anti-inflammatory effect of PRD associated with the antineoplastic effect of ETO.

**Fig. 9:**
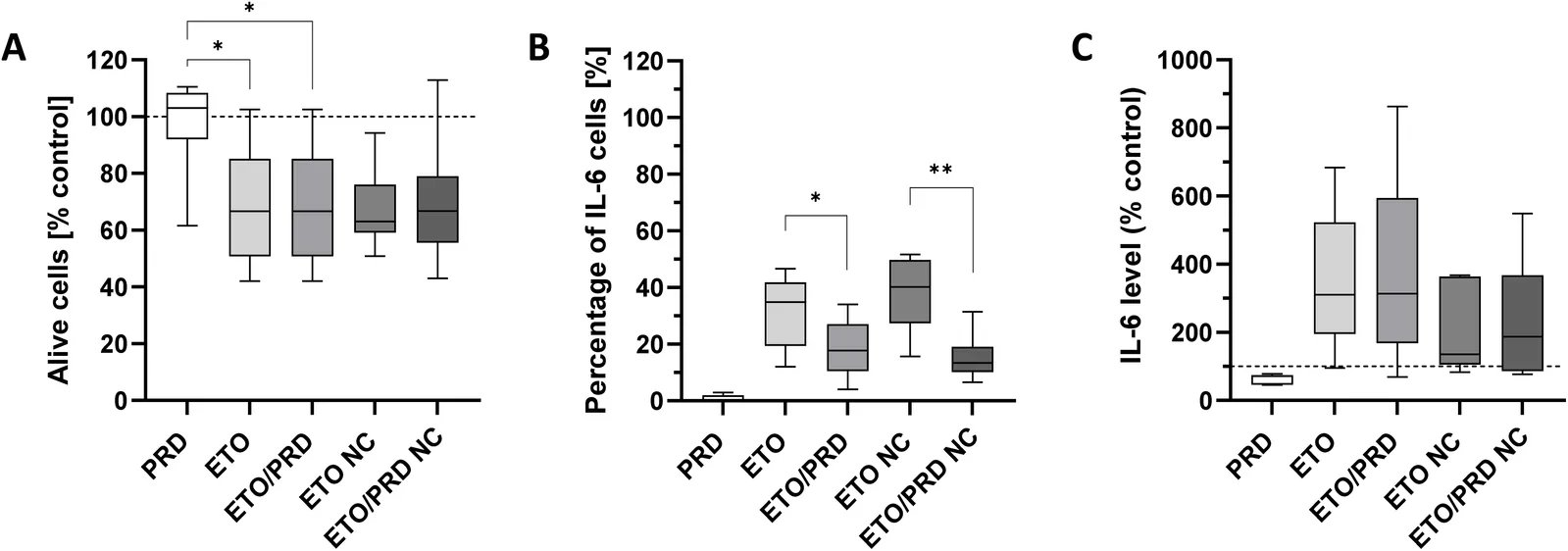
Percentage of alive cells (A) and IL-6 positive cells (B) after incubation with different formulations evaluated by flow cytometry. Supernatant IL-6 level for the similar condition was evaluated by ELISA (C). Mean ± SEM, Mann Whitney test.

## Conclusions and future perspectives

The concept of combination therapy involves the integration of the most advantageous properties of both compounds to achieve a synergistic effect, thereby optimizing their therapeutic potential. To that end, we have successfully formulated etoposide/prednisolone co-nanocrystals using a bottom-up approach with better stability than the prednisolone nanocrystals, which were unstable as mono-nanocrystals. Hence, this study reports for the first time the preparation of ETO/PRD co-NCs based on the solvent/antisolvent nanoprecipitation method. The freshly prepared co-NCs present an average size of 191.9 ± 6.5 nm, a relatively low polydispersity index of 0.28 ± 0.09, and are neutral (zeta potential of −5.0 ± 0.7 mV). The drug loading was 72% ± 1% for PRD and 84% ± 4% for ETO in the ETO/PRD co-NCs. DLS, NTA, and TEM were complementary methods used to characterize mono- and co-NCs by comparing their size and morphology. Furthermore, poloxamer P407 plays a major role in stabilizing the nanocrystals. Noteworthily, it has been shown that the presence of ETO/PRD in the co-NC formulation improves the PRD stabilization as NCs. In addition, the NCs showed colloidal stability for at least 70 days at 4 °C. In the release experiments, both active pharmaceutical ingredients showed a similar release kinetics.

Importantly, the co-NCs exhibited a strong anticancer activity *in vitro*, as assessed by cytotoxicity experiments. The anticancer efficiency of the ETO/PRD co-NCs was significantly higher than that of the ETO NCs or the free ETO formulation. Additionally, the ETO/PRD co-NCs demonstrated enhanced anti-angiogenic properties on endothelial cells, as determined by capillary tube formation assays, and increased inhibition of cell migration during the scratch healing assay. The anti-angiogenic effect was further confirmed through VEGF ELISA immunoassays, which showed that the co-NCs retained their ability to inhibit VEGF production compared to the mono-NCs and the related free API mixtures after 3 days incubation with endothelial cells.

The *in vitro* results of the ETO/PRD co-NCs in this study are promising for use in synergistic biopharmaceutical applications. The use of mixed nanocrystals for co-delivering PRD and ETO demonstrated synergistic chemotherapeutic effects and stronger anti-angiogenic and antimigratory effects compared to those of individual free drugs or their mixtures. These findings suggest that the co-NCs have the potential to be a novel nanomedicine system, offering advantages such as high drug loading and stable co-delivery of organic drug combinations, which can contribute to significant advances in cancer therapy compared to traditional organic NCs.

Overall, the findings of this research demonstrate the efficacy of nanocrystallization in the formulation of innovative mixed APIs, resulting in enhanced stability and/or biological efficiency. The results obtained provide a foundation for further optimization of various pharmaceutical co-preparations, especially for optimizing cancer treatments. To date, only few papers have dealt with co-NCs, with no studies examining their therapeutic effects, notably for antiretroviral APIs^56^ and the mixed flavonoid/vitamin B3 system.^57^ Moreover, the first NC formulation approved by the FDA and the European Medicines Agency authorities with two different drugs, in 2021 and 2022, respectively, is Cabenuva®, which is administrated by the iv route through two different injections as the two antiretroviral APIs, namely, cabotegravir and rilpivirine, could not be stabilized in a unique dosage form.^58^

## Author contributions

Panpan Ma: investigation (formulation, characterization, and optimization of the nanocrystals, and *in vitro* experiments), formal analysis, writing – original draft, reviewing, and editing. Abdelrahman Hassan: investigation (formulation, characterization, and *in vitro* experiments), and reviewing. Sarah Diakhaby: investigation (*in vitro* experiments) and reviewing. Syed Nafis Shadman Ali: investigation (formulation, characterization, and *in vitro* experiments) and reviewing. Luis Castillo Henriquez: investigation (formulation and characterization of the nanocrystals). Marianne Bombled: investigation (HPLC experiments). Charlotte Izabelle: investigation (TEM experiments). Libor Kostka: investigation (NTA experiments). Tomáš Etrych: reviewing. Rabah Gahoual: investigation (HPLC experiments). Brice Martin: reviewing. Johanne Seguin: conceptualization and methodology (*in vitro* experiments), investigation (*in vitro* experiments), formal analysis, writing – original draft, reviewing and editing. Nathalie Mignet: reviewing and editing. Yohann Corvis: conceptualization, methodology, investigation, and validation (formulation, characterization, and optimization of the nanocrystals), formal analysis, writing – original draft, reviewing and editing.

## Conflicts of interest

There are no conflicts of interest to declare.

## Supplementary Material

NA-008-D5NA00941C-s001

## Data Availability

The data supporting this article have been included as part of the supplementary information (SI). Supplementary information is available. See DOI: https://doi.org/10.1039/d5na00941c.
