# Supplementary material for: Engineered etoposide-prednisolone co-nanocrystals for synergistic antitumor efficacy enhancement
Source: Nanoscale Adv. 2026 Jul 1;8(20):5838–49. doi: 10.1039/d5na00941c (PMC13439618; doi:10.1039/d5na00941c)
Supplement: Supplementary file 1 [file NA-008-D5NA00941C-s001.pdf]

Supplementary Information (SI) for Nanoscale Advances.
This journal is © The Royal Society of Chemistry 2026

Supplementary Information

Engineered Etoposide-Prednisolone Co-nanocrystals for Synergistic Antitumor Efficacy Enhancement

Panpan Ma[a,b], Abdelrahman Hassan[a], Sarah Diakhaby[a], Syed Nafis Shadman Ali[a], Luis Castillo Henriquez[a], Marianne Bombled[a], Charlotte Izabelle[c], Libor Kostka[d], Tomáš Etrych[d], Rabah Gahoual[a], Brice Martin[a,e], Johanne Seguin[a], Nathalie Mignet[a] and Yohann Corvis[a*]

[a] Université de Paris, CNRS, Inserm, UTCBS, Chemical and Biological Technologies for Health Group (https://utcbs.u-paris.fr/en), Faculté de Pharmacie, 4 avenue de l'Observatoire, 75006 Paris, France.
[b] Department of Radiology, Lucas Center, 1201 Welch Road Stanford University, Stanford, CA, USA.
[c] Université Paris Cité, UAR3612 CNRS, US25 INSERM, Cellular and Molecular Imaging Facility, 75006 Paris, France
[d] Institute of Macromolecular Chemistry, Czech Academy of Sciences, Heyrovského náměstí 2, Prague, CZ-162 06 Czech Republic
[e] Department of Neurological Surgery, Weill Medical College of Cornell University, New York, NY, USA.

Correspondence and requests for materials should be addressed to Y.C. (yohann.corvis@u-pariscite.fr)

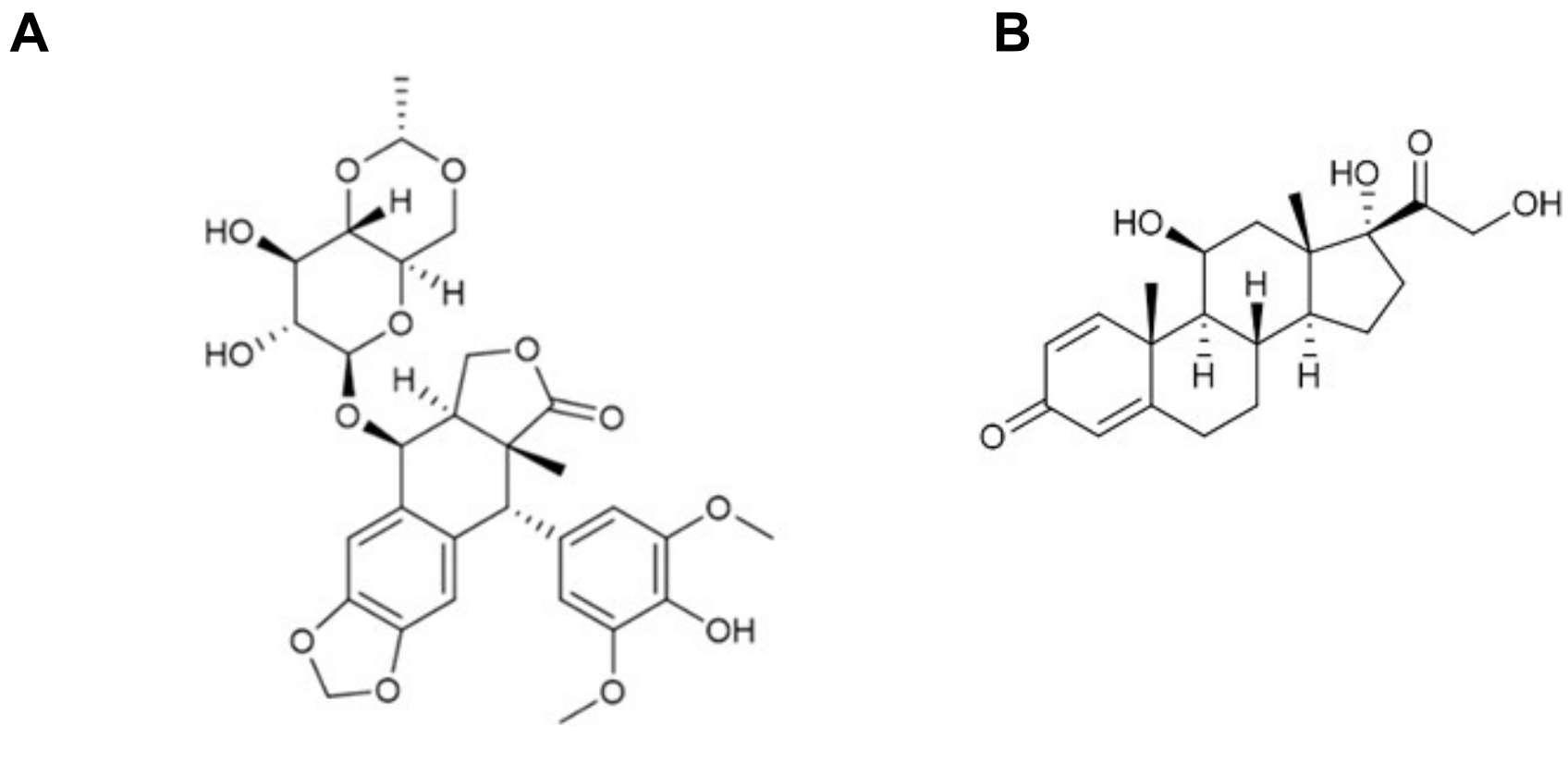

Fig. S1 Chemical structure of Etoposide (A) and Prednisolone (B).

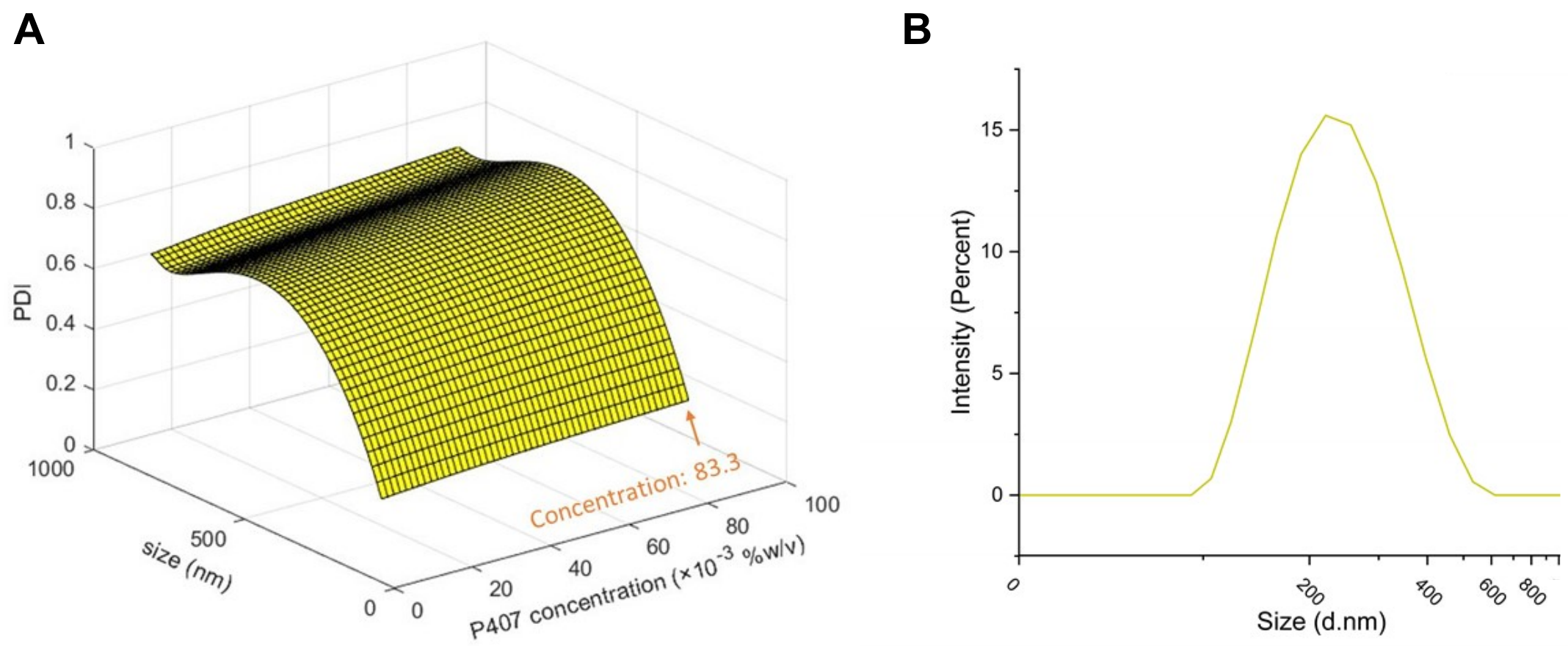

Fig. S2 (A) Size and PDI of Prednisolone NCs stabilized with different concentrations of P407 measured by diffusion light scattering. Data was processed with MATLAB 2021a. (B) A DLS size distribution data for ETO NCs.

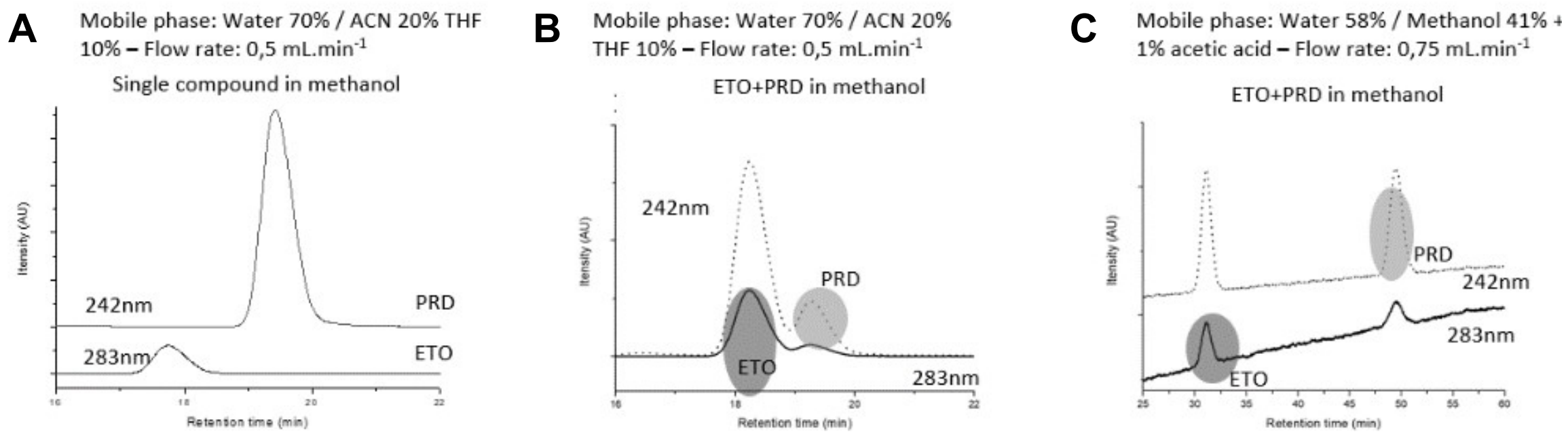

Fig. S3 (A-C) Optimization of different mobile phases. (A-B) Retention time and chromatogram intensity of PRD and ETO with 70% water /ACN 20%/THF 10%. mobile phase. (C) Retention time and chromatogram intensity of PRD and ETO with 1% acetic acid and 41% MeOH in milli-Q water (solid line λ_{283nm}, dotted line λ_{242nm}).

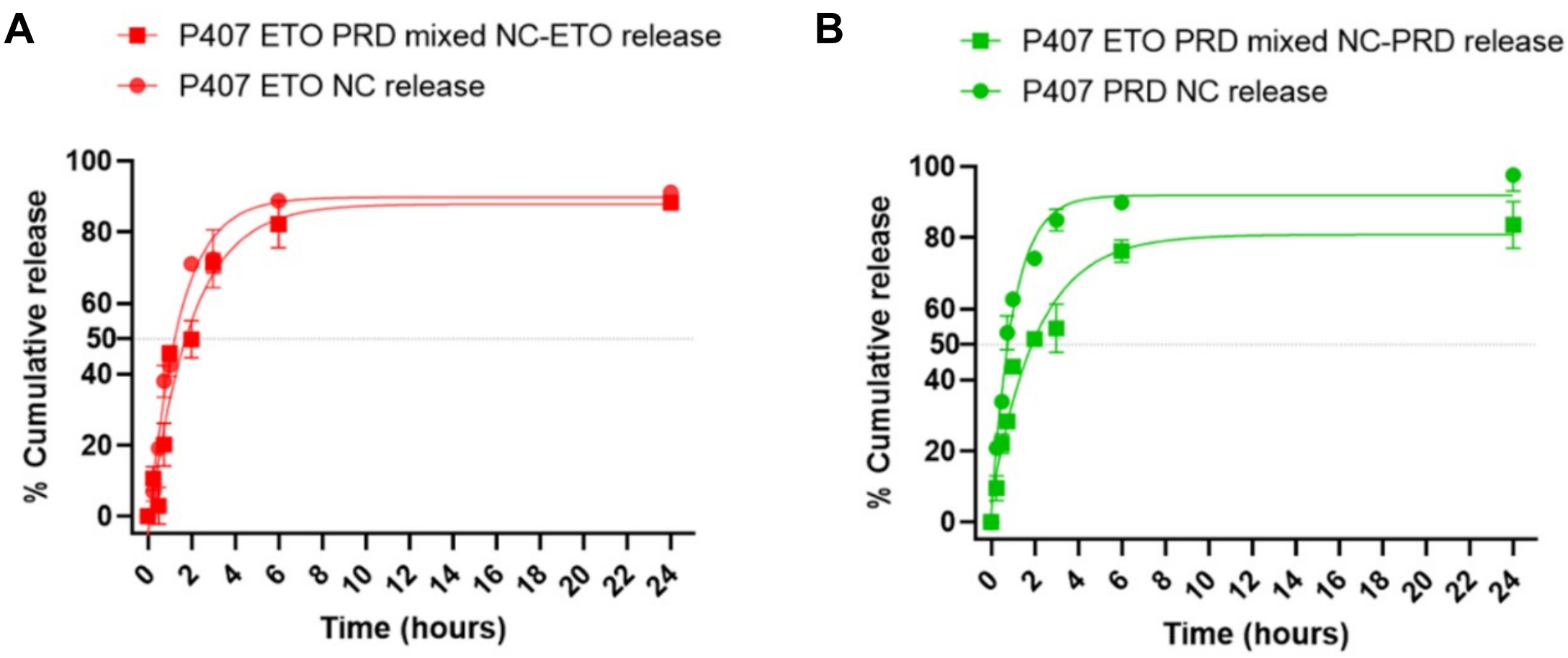

Fig. S4 First-order release kinetics profiles of ETO release from mixed NCs and single NCs (A), PRD release from mixed NCs and single NCs (B) in HBS incubated at 37 °C under shaking for 24 hours (n = 3). IC50 for various formulations were labeled in each figure. The data were processed with GraphPad Prism version 9 and represent the means ± SD of three independent experiments.

Table S1 Hydrodynamic size (in intensity) and PDI (value ± SD) of NCs suspension in water, stored at 4 ℃, using dynamic light scattering, n = 3

**Time (day)**	**Nanocrystal sample**					
	**PRD NCs**		**ETO NCs**		**PRD/ETO NCs**	
	Size (nm)	PDI	Size (nm)	PDI	Size (nm)	PDI
**0**	158.9 ± 4.7	0.22 ± 0.03	249.7 ± 4.8	0.25 ± 0.03	191.9 ± 6.5	0.28 ± 0.09
**1**	173.7 ± 5.0	0.17 ± 0.03	250.3 ± 1.6	0.13 ± 0.02	251.9 ± 5.2	0.23 ± 0.05
**3**	171.6 ± 3.0	0.24 ± 0.07	250.2 ± 6.1	0.29 ± 0.01	251.3 ± 4.1	0.24 ± 0.01
**30**	175.4 ± 8.8	0.28 ± 0.06	245.2 ± 8.6	0.15 ± 0.02	265.7 ± 9.0	0.24 ± 0.02
**60**	183.7 ± 6.9	0.21 ± 0.07	243.7 ± 7.7	0.21 ± 0.03	281.8 ± 4.8	0.22 ± 0.03

Table S2 Different mobile phase screening used for HPLC testing

**Mobile phase**	**Flow rate (mL.minute^{-1})**	**Retention time**	
		PRD	ETO
1% acetic acid, 41% MeOH and 58% Milli-Q water	0.75	50	30
70% water /CAN 20%/THF 10%	0.5	15.5	17.9
